# Is the worst of the COVID-19 global pandemic yet to come? Application of financial mathematics as candidate predictive tools

**DOI:** 10.1038/s41398-021-01429-0

**Published:** 2021-05-20

**Authors:** Prashant Dogra, Eugene J. Koay, Zhihui Wang, Farhaan S. Vahidy, Mauro Ferrari, Renata Pasqualini, Wadih Arap, Marc L. Boom, H. Dirk Sostman, Vittorio Cristini

**Affiliations:** 1 Mathematics in Medicine Program, Houston Methodist Research Institute, Houston, TX USA; 2 Department of Gastrointestinal Radiation Oncology, The University of Texas MD Anderson Cancer Center, Houston, TX USA; 3 Center for Outcomes Research, Houston Methodist Research Institute, Houston, TX USA; 4 Houston Methodist Neurological Institute, Houston Methodist, Houston, TX USA; 5 Department of Pharmaceutics, University of Washington, Seattle, WA USA; 6 Dompé X-Therapeutics, San Mateo, CA USA; 7 Rutgers Cancer Institute of New Jersey, Newark, NJ USA; 8 Department of Radiation Oncology, Division of Cancer Biology, Rutgers New Jersey Medical School, Newark, NJ USA; 9 Department of Medicine, Division of Hematology/Oncology, Rutgers New Jersey Medical School, Newark, NJ USA; 10 Department of Medicine, Houston Methodist, Houston, TX USA; 11 Weill Cornell Medicine, New York, NY USA; 12 Houston Methodist Research Institute, Houston, TX USA; 13 Houston Methodist Academic Institute, Houston, TX USA

**Keywords:** Human behaviour, Diseases

## Abstract

The Elliott Wave principle is a time-honored, oft-used method for predicting variations in the financial markets. It is based on the notion that human emotions drive financial decisions. In the fight against the COVID-19 global pandemic, human emotions are similarly decisive, for instance in that they determine one’s willingness to be vaccinated, and/or to follow preventive measures including the personal wearing of masks, the application of social distancing protocols, and frequent handwashing. On this basis, we postulated that the Elliott Wave Principle may similarly be used to predict the future evolution of the COVID-19 pandemic. We demonstrated that this method reproduces the data pattern for various countries and the world (daily new cases). Potential scenarios were then extrapolated, from the best-case corresponding to a rapid, full vaccination of the population, to the utterly disastrous case of slow vaccination, and poor adherence to preventive protocols.

## Introduction

In 1938, Ralph Nelson Elliott, an accountant by profession, argued that the stock market is a phenomenon governed by crowd psychology^1^. In his classic books *The Wave Principle*^1^ and *Nature’s Law: The Secret of the Universe*^2^, Elliott described that human emotions, which tend to be rhythmical and follow patterns of optimism and pessimism, are instrumental in shaping market dynamics. The sentiments of the crowd can lead to quantifiable patterns in human activities, and when applied to financial markets, such patterns are observed in the movement of asset (stocks, commodities, etc.) prices across time scales ranging from hours to years. These observations made by Elliott lead to his description of the Wave Principle, which is used by traders for *technical analysis* to understand market behavior and predict future trends^3^. Given the ability of Elliott Wave Principle to describe human sentiment-driven activities, we hereby hypothesized that the Wave Principle could also describe the epidemiological evolution of the COVID-19 global pandemic.

Our hypothesis can be contextualized through a simple thought experiment. Imagine two healthy individuals A and B of the same age, living in a common geographical location with prevalence of COVID-19 cases, such that public health policies related to gatherings, masks, business closures, and social distancing are in effect. While individual A strictly and unchangingly obeys the policies, individual B tends to be more casual and is influenced by external or internal factors (such as fluctuations in daily new cases or the need to socialize and be outdoors) in their adherence to the policies. Simply put, individual A follows the guidelines *objectively* as long as they are in place, while their counterpart is more amenable to their *subjective choice*, driven primarily by feelings, in following the guidelines. Although biologically, the two individuals maybe equally prone to infections, but behaviorally speaking, it can be safely argued that individual A and their likes are less prone to infections during a pandemic, while individuals like B tend to be more vulnerable and thus fuel the growth of the pandemic. With this premise, we investigated the ability of the sentiment-driven Elliott Wave Principle in describing the evolution of the COVID-19 global pandemic. To the best of our knowledge, the current work is the first ever application of Elliott Wave Principle to the study of epidemiology of viral infections at the global or regional scale.

## Methods

### The basics of Elliott Wave Principle

**Fig. 1:**
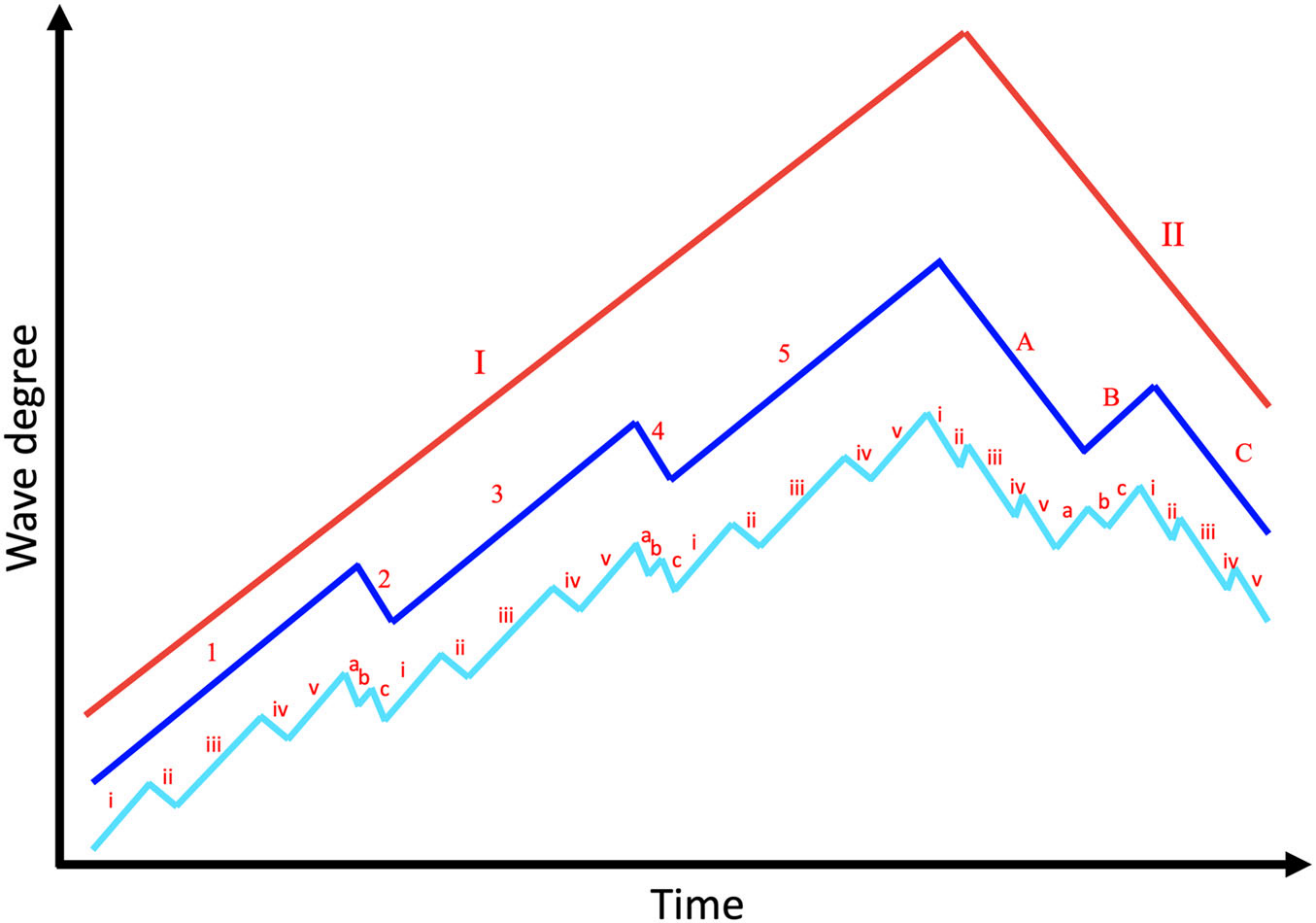
Elliott waves of smallest degree or dimension (sub-waves; cyan) are shown along with the waves of higher degree (waves, blue; super-waves, red). i, iii, v are impulses of the smallest dimension; 1, 3, 5 are impulses of next higher dimension; I is the impulse of next higher dimension. Similarly, ii and iv are correctives of the smallest dimension, 2 and 4 are correctives of the next higher dimension, and II is the corrective of next higher dimension. Impulses (e.g., I) are made up of 5 waves of lesser dimension (i.e., 1, 2, 3, 4, 5), while correctives (e.g., II) are made up of 3 waves of lesser dimension (i.e., A, B, C).

In brief, the Wave Principle states that movement of asset prices occurs on a wave, where a completed movement is made up of five sub-waves. As shown in Fig. 1, sub-waves i, iii, and v (cyan lines) are in the direction of movement (known as *impulses*), while sub-waves ii and iv (cyan lines) are contrary to the direction of movement (known as *correctives*). Elliott identified a fractal character in the waves of asset prices, such that the five sub-waves of one dimension or degree (i.e., sub-waves i, ii, iii, iv, v) become the first wave of the next higher dimension (i.e., wave 1), and so on. Notably, impulses and correctives differ from each other such that the former are divisible into five sub-waves of lesser dimension (i to v), while the latter are divisible into three sub-waves of lesser dimension (a, b, c). The impulsive and corrective moves of an Elliott wave follow *Fibonacci ratios*^4^, which are mathematical relationships between the numbers of the Fibonacci sequence^1^. The various waves of an Elliot wave relate to each other through Fibonacci ratios and thus the ratios are used to predict the extensions and retracements in asset prices, a technique commonly known as the *Fibonacci Pinball*^5^. Since the wave principle is based on sentiments of the crowd, we reasoned that other fields of human activity involving masses might perhaps behave in a fashion predictable by the Wave Principle.

### Fibonacci pinball

To perform the Elliott Wave analysis, Fibonacci retracements and Fibonacci extensions are calculated from the current and past prices of the asset (daily infections in our case) to project its future price, based on the commonly observed Fibonacci ratios (Fib). These retracements and extensions provide support and resistance levels, where a support level indicates the price below which assets do not fall and resistance level represents the price above which assets do not rise for a period of time, to forecast the price movement of the asset over time. Generally, six such levels are generated to identify possible support and resistance levels for an asset and set price targets during trading.

To calculate the new price (*P*_*t*+1_) via a Fibonacci retracement, the price of the asset at the last two timepoints (*P*_*t*_ and *P*_*t*−1_) is used in the following formulae:

During uptrend (Bullish market):

$$P_{t + 1} = P_t - \left( {P_t - P_{t - 1}} \right) \cdot {\mathrm{Fib}}$$

During downtrend (Bearish market):

$$P_{t + 1} = P_t + \left( {P_{t - 1} - P_t} \right) \cdot {\mathrm{Fib}}$$

To calculate the new price (*P*_*t*+1_) via a Fibonacci extension, the price of the asset at the last three timepoints (*P*_*t*_, *P*_*t*−1_, and *P*_*t*−2_) is used in the following formulae:

During uptrend (Bullish market):

$$P_{t + 1} = P_t + \left( {P_{t - 1} - P_{t - 2}} \right) \cdot {\mathrm{Fib}}$$

During downtrend (Bearish market):

$$P_{t + 1} = P_t - \left( {P_{t - 2} - P_{t - 1}} \right) \cdot {\mathrm{Fib}}$$

The data used for analyses was obtained from a public database available here:^6^ https://github.com/owid/covid-19-data/blob/master/public/data/jhu/new_cases.csv. All analyses were performed in MATLAB R2018a. The computational code written to conduct the analyses and generate the plots is available from the corresponding author upon reasonable request.

## Results and discussion

### Applying the Wave Principle to the COVID-19 pandemic

Given that more than 124 million people have been infected and more than 2.7 million patients have died worldwide due to COVID-19 as of March 2021^7^, human emotions have thus been running particularly high during the pandemic. Furthermore, especially with the shutdown of economies and social isolation due to travel restrictions and lockdowns, human emotions have been going back and forth while adjusting to “the new normal.” Against this backdrop, we have looked at the 7-day moving average of daily new cases of COVID-19 disease, in hard-hit countries/regions, since the beginning of the pandemic^6^.

**Fig. 2:**
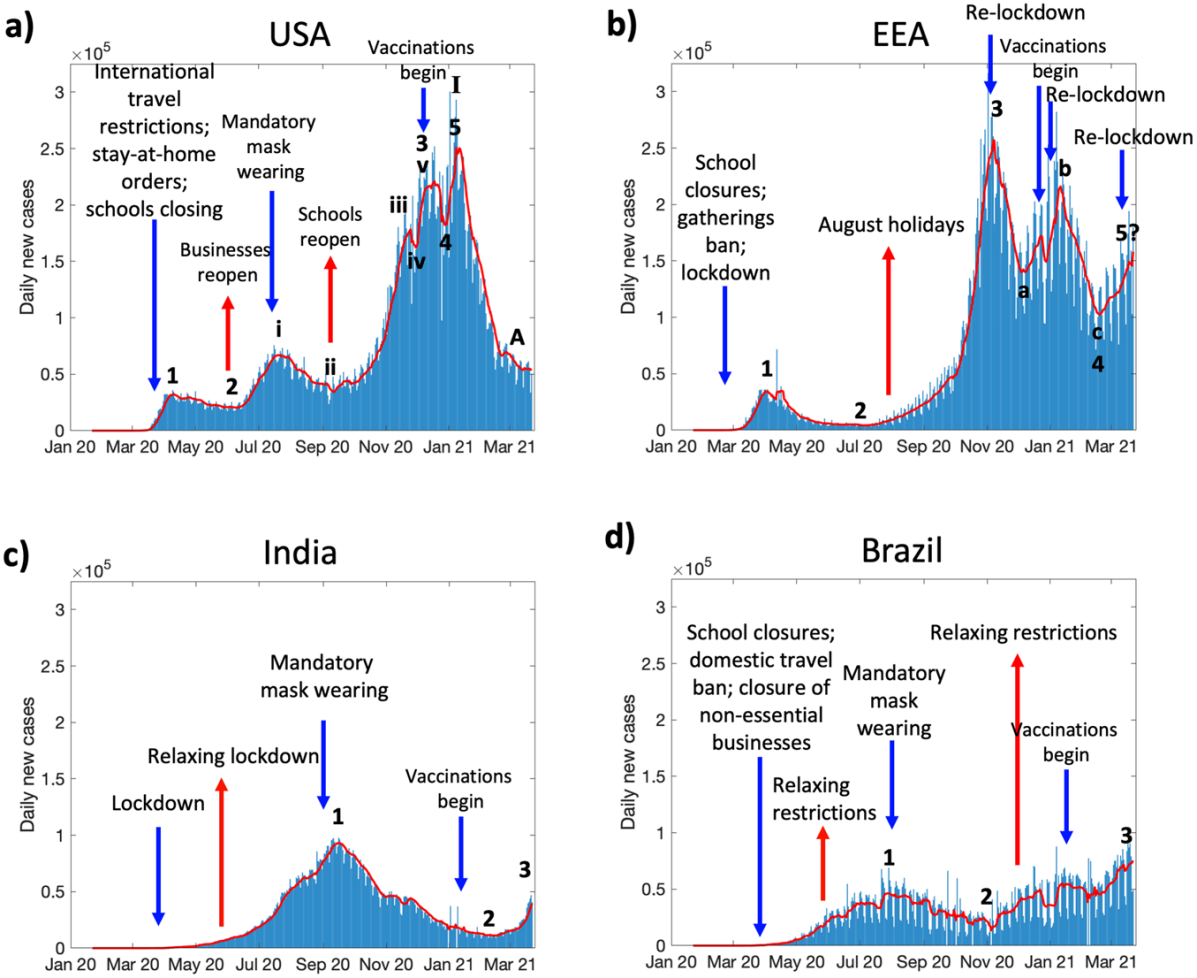
Application of Wave Principle to COVID-19 pandemic. Daily new case timeline of COVID-19 in (**a**) USA, **b** European Economic Area (EEA), **c** India, and (**d**) Brazil, until 3/22/2021. Red line represents 7-day moving average of the cases. Impulses 1, 3, 5, (or, i, iii, v) and correctives 2 and 4 (or, ii and iv) are shown wherever they are visible. Blue and red arrows indicate the major restrictive and permissive events, respectively. Data source: https://github.com/owid/covid-19-data/blob/master/public/data/jhu/new_cases.csv.

Indeed, as shown in Fig. 2a, the trend for USA seems to be obeying the Wave Principle on a timescale of weeks, i.e., it exhibits an initial impulse (wave 1), followed by a corrective (wave 2), and then an extended impulsive wave 3, which is made up of 5 sub-waves of smaller dimension (i.e., sub-waves i, ii, iii, iv, and v). Following which a corrective wave 4 and an impulsive wave 5 bring the high dimensional impulsive super-wave I to completion. The current trend (as of 3/22/2021) appears to be in a corrective wave A of II. This pattern can be attributed to crowd psychology that manifested itself in the form of a rhythmical pattern of adherence to public health policies imposed by the government, due to fear of infection, followed by either relaxation of policies by the government, or disobedience to policies by the masses, due to economic turmoil and growing resentment. As currently evident, the imposition of international travel restrictions, stay-at-home orders, and closure of schools and businesses due to growing number of cases around mid-March 2020, followed by mandatory use of masks in public places starting mid-July 2020, largely promoted the corrective waves (during April and May 2020 (wave 2) and August 2020 (sub-wave ii), respectively). However, these correctives may have given the masses a false sense of the infection “being over with” and leading into them becoming less vigilant. Also, the growing unrest in public due to closure of businesses and plummeting GDP in the second quarter of 2020 has led to relaxation of some policies by the government, particularly opening of businesses like restaurants, bars, and gyms in early June 2020, and then schools in early September 2020, thereby instigating the impulses thereafter (sub-wave i in late June and July 2020 and sub-wave iii in October and November 2020, respectively). Of note, the third wave of an Elliott wave is typically the largest and the most powerful, which is also evident above in the observation that wave 3 has brought about an increase in daily cases by one order of magnitude compared to the value at the base of wave 2, thereby adhering to the wave principle. Similarly, the smaller dimensional sub-wave iii is also the strongest impulse of wave 3.

Many other countries seem to be obeying the principle, although none seem to have completed super-wave I (higher dimension) yet. For instance, after an initial impulsive wave 1 (Fig. 2b), the European Economic Area (EEA, comprising of 32 European countries) had an extended corrective wave 2 following stringent restrictions starting March 2020 that lasted through summer 2020. The extended corrective wave must have made the crowds less watchful during the European holiday season in August 2020, following which a strong impulsive wave 3 was seen. This surge was corrected by multiple regional lockdowns and beginning of vaccinations in most European nations leading to the corrective wave 4 that was made up of sub-waves a, b, and c. However, a recent uptick since late February 2021 due to loosened restrictions and slow rate of vaccinations appears to have initiated an impulsive wave 5, which in the best-case scenario will fail and be truncated by renewed regional lockdowns and higher vaccination rates, but may cross the peak of wave 3 in the worst-case scenario to complete the impulsive wave I in EEA.

Further, India (Fig. 2c) and Brazil (Fig. 2d), the other two most hard-hit countries, imposed restrictions early-on in the pandemic during March 2020 that lasted around 8 weeks, which helped delay impulsive wave 1 in these countries. But, after the easing of restrictions in June 2020 due to declining economy and growing unrest in the masses, impulsive wave 1 took off. While no new nationwide restrictions were then imposed, but due to other measures like mandatory use of masks in public places and suggestive social distancing, a corrective wave 2 followed. While Brazil is exhibiting an impulsive wave 3 that can be attributed to the revival of tourist attractions, beaches, shopping malls, and restaurants since October 2020^8^, India seems to have been on a prolonged correction that appears to be dying only recently. It is important to note that in case of India, there is growing evidence that the Indian population possesses better innate immunity to SARS-CoV-2^9–11^, which indicates that any possible divergence from the sentiment-mediated wave principle maybe a result of the additional biological variable at play in this population.

### The crisis may continue in 2021

Since mid-December 2020, vaccines started to become available hierarchically, based on the susceptibility of populations, in high-income countries. However, due to logistical limitations, vaccines may not be available to the larger population anytime soon^12–14^, but irrespective of that, based on a survey published by Pew Research Center, *39% of the US adults have expressed lack of intent to get a vaccine, and 62% express being uncomfortable to be the first ones to get the vaccine*^15^. On the other hand, the majority of the population (~80%) in upper- (e.g., Brazil) and lower-middle (e.g., India) income countries may not receive the vaccine until the end of 2021^16^. Moreover, the recent emergence of variants of SARS-CoV-2 in the UK and South Africa, capable of increasing the reproduction number (R_0_) of COVID-19, are concerning for their increased infectivity and transmissibility and the potential to cause vaccine failure^17–19^. While SARS-CoV-2 mutates less frequently than influenza viruses, and breakthrough infections due to vaccine failure have not yet been registered, but mutations over time may require alterations in vaccines, thereby slowing down the attainment of herd immunity^20^. Thus, for the above reasons, one may conjecture that if effective vaccines are not administered globally to the masses anytime soon, the pandemic will inevitably exhibit higher-dimensional waves II, III, IV, and V over the coming year or two. This will potentially lead to a massive rise in new infections and deaths, unless the socio-medical parameters are changed by permanent adherence to stringent policies *and* introduction of vaccines readily available to the larger population.

**Fig. 3:**
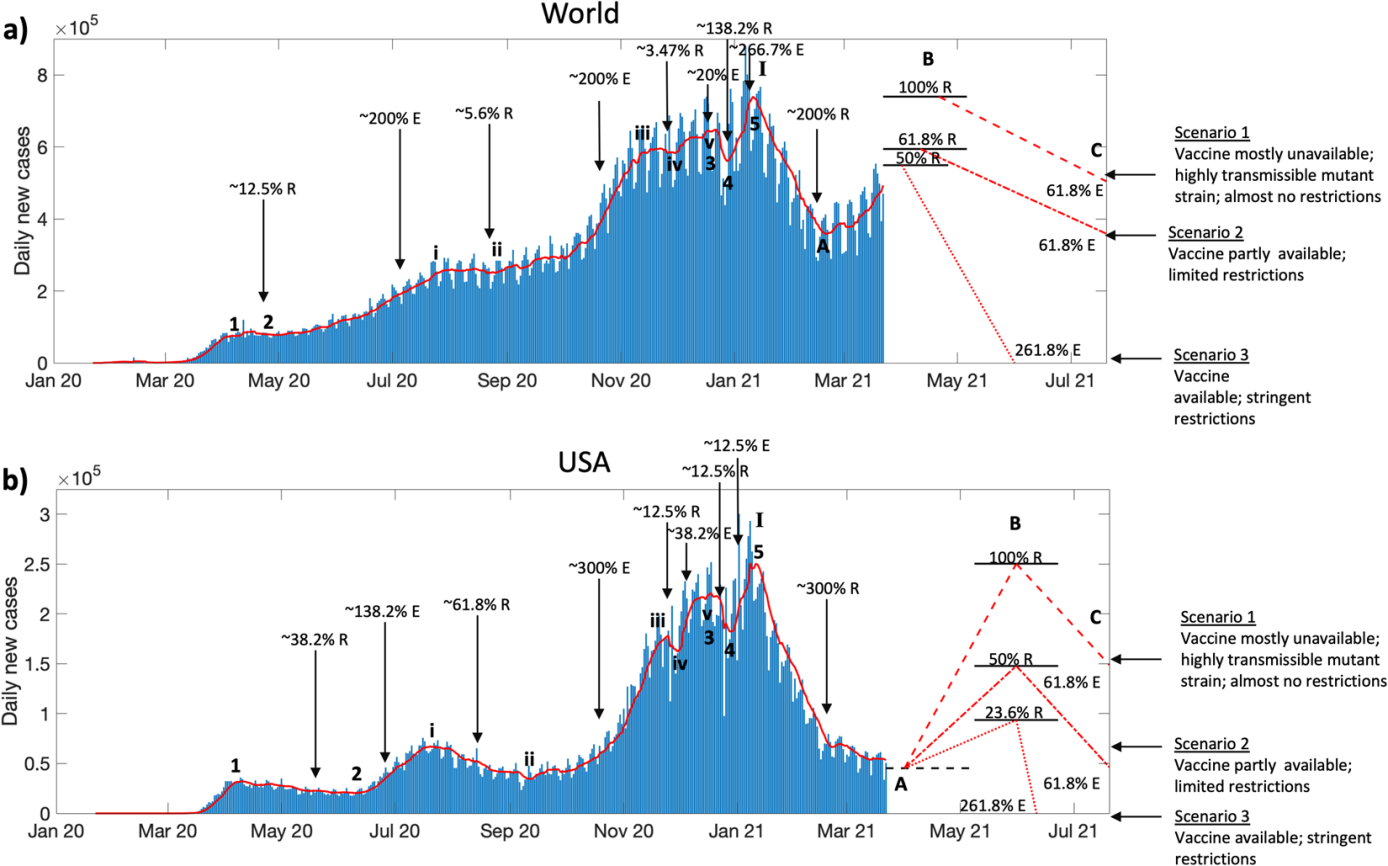
Predictions based on the Wave Principle. Elliott wave analysis for the (**a**) world and (**b**) USA using the Fibonacci Pinball method. Dotted, dashed-dotted, and dashed red lines show predictions of best-, intermediate-, and worst-case scenarios, respectively, for daily new infections. Black arrows point out the closest Fibonacci ratio exhibited by an extension (E) or a retracement (R) of the wave. Horizonal black solid and dashed lines represent the resistance and support levels, respectively, for daily new cases based on the most common Fibonacci ratios.

By using the Fibonacci Pinball method of Elliott Wave analysis^4,21^, we can make projections for how the daily cases will evolve over time in the coming year, and we demonstrate these projections for the world and USA. As shown in Fig. 3, the retracements (R) and extensions (E) assumed by the waves thus far approximately adhere to Fibonacci ratios, thereby suggesting that the pandemic has adhered to the Wave Principle and it is reasonable to use Fibonacci Pinball to make future projections. The current impulsive wave B of super-wave II in the world (Fig. 3a) is undergoing a downtrend retracement that will be resisted at one of the several retracement levels (shown here 50%, 61.8%, and 100% R levels), such that a downtrend extension will then follow, which will further correct the wave. Based on the level achieved by the current impulse B, the downtrend extension can take one of the several possible levels such that mild, moderate, and strong correction will be achieved by the end of super-wave II in August 2021. This outcome will be determined by the mass availability of vaccines globally and the adherence to public health policies before immunization can be achieved.

Similarly, the USA currently appears to be on a corrective wave A of super-wave II (Fig. 3b), which may face support at the 300% R level, following which an impulsive wave B will cause a downtrend retracement to achieve one of the several possible retracement levels (shown here are 23.6, 50, and 100% R). The impulse B can be attributed to relaxation of policies, especially unconditional opening of schools and businesses in several states, the spring break gatherings and celebration, and the slow rates of nationwide vaccination. Eventually, the impulse B can be expected to correct itself through wave C, possibly due to renewed restrictions in the face of rising cases, or improvement in vaccination rates in the coming months due to availability of new vaccines, to achieve support at one of the several possible downtrend extension levels. Also, based on the vaccination rates and adherence to policies, a mild, moderate, or strong correction can be expected from wave II.

Following the completion of wave II, an impulsive rally of wave III may show attainment of unprecedented levels of infection in the world and/or USA, or may alternatively fail at a lower resistance level, depending upon the mass availability of vaccines globally and adherence to public health policies in the second half of 2021. Thus, as shown in Fig. 3, if our estimates are correct, then *this pandemic is here to stay through 2021*. As a representative example, if current policies continue in the United States as they did in 2020, wave III will likely last the better part of 2021 and affect the vast majority of the US population (note that this is typical for such one-in-a-century pandemic events). While the Biden administration is expected to change current federal approaches to the pandemic, adoption of these policies uniformly throughout the United States is expected to be challenging due to sociopolitical resistance. In the absence of a widely distributed and effective vaccine *as well as* stringent public health policies throughout, the pandemic may last throughout 2021 in the USA.

## Conclusions

**Fig. 4:**
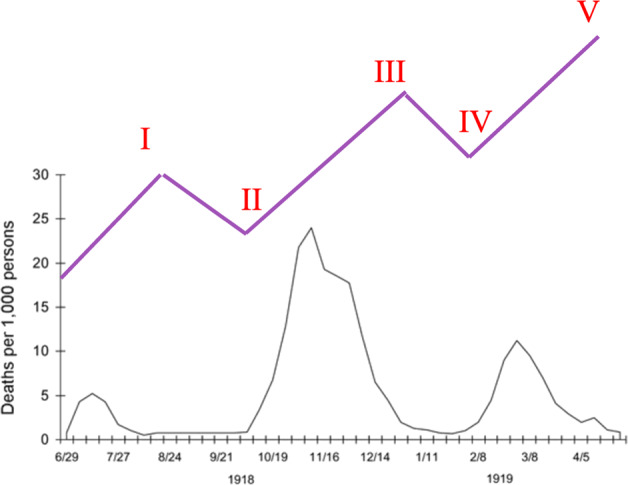
Three cycles of illness during pandemic influenza (Spanish flu) of 1918–1920 (black). Hypothetical Elliott wave (purple) is overlaid on the plot to demonstrate correspondence of deaths with different waves of the pandemic. Adapted from: https://www.cdc.gov/flu/pandemic-resources/1918-commemoration/three-waves.htm.

While China was presumably the first place to be hit by COVID-19, but regardless it was able to control the infection swiftly and efficiently due to the existence of a centralized epidemic response system, which also gained support from the masses due to the mental preparedness of adults who had witnessed the fatality of SARS-CoV epidemic in 2002–2003^22^. It is thus evident that human emotions act as the fuel for the fury of a pandemic, and thus without *both* stringent public health policies and availability of a vaccine to the larger population to achieve herd immunity, it will be virtually impossible to control the pandemic. Thus, it will not be wrong to speculate that the growing pandemic fatigue among the masses will make the public lower its guard against SARS-CoV-2, and the additional absence of a vaccine is a recipe for disaster that is waiting to unfold itself to extend the pandemic deep into 2021. Historically, the H1N1 influenza pandemic (also called Spanish flu) of 1918–1920, which is one of the most severe pandemics of history, occurred in three major cycles of illness^23^ and caused deaths of ~50 million people globally, with 675,000 in the United States alone^24^. Since the case-fatality ratio of Spanish flu was ~2.5% and the population of USA in 1918 was ~103 million, therefore ~27 million Americans must have contracted the infection^25^. Assuming that the same fraction of population will be infected by COVID-19, it can be estimated that potentially *~85 million Americans will be infected by the pandemic, and with a case-fatality ratio of 1.8% in the USA*^26^*, ~1.5 million deaths can occur*. Note that with the availability of more FDA-approved therapeutics for COVID-19, the case-fatality ratio is expected to go down further, thereby decreasing fatalities. The three cycles of Spanish flu deaths are equivalent to one complete movement of the Elliott wave, i.e., the three major death events correspond to waves I, III, and V (impulses) of an Elliott wave (Fig. 4), out of which wave III was the deadliest, thereby lending further evidence to our predictions. *Thus, to win over the COVID-19 pandemic effectively, it is critical to empower the corrective super-wave II by effective implementation of public health policies, even in the face of decreasing infections, to buy time for mass production of vaccines*.

## Footnotes

1 In a Fibonacci sequence, each number is the sum of the two preceding numbers. Thus, the sequence is: 0, 1, 1, 2, 3, 5, 8, 13, 21, 34, and so on.
